# Skill Set or Mind Set? Associations between Health Literacy, Patient Activation and Health

**DOI:** 10.1371/journal.pone.0074373

**Published:** 2013-09-04

**Authors:** Samuel G. Smith, Laura M. Curtis, Jane Wardle, Christian von Wagner, Michael S. Wolf

**Affiliations:** 1 Health Behaviour Research Centre, Department of Epidemiology and Public Health, University College London, London, United Kingdom; 2 Health Literacy and Learning Program, Division of General Internal Medicine and Geriatrics, Feinberg School of Medicine at Northwestern University, Chicago, Illinois, United States of America; 3 Department of Learning Sciences, School of Education and Social Policy, Northwestern University, Evanston, Illinois, United States of America; Iran University of Medical Sciences, Islamic Republic of Iran

## Abstract

**Objective:**

There is ongoing debate on whether health literacy represents a skill-based construct for health self-management, or if it also more broadly captures personal ‘activation’ or motivation to manage health. This research examines 1) the association between patient activation and health literacy as they are most commonly measured and 2) the independent and combined associations of patient activation and health literacy skills with physical and mental health.

**Methods:**

A secondary analysis of baseline cross-sectional data from the LitCog cohort of older adults was used. Participants (n = 697) were recruited from multiple US-based health centers. During structured face-to-face interviews, participants completed the Test of Functional Health Literacy in Adults (TOFHLA), the Patient Activation Measure (PAM), the SF-36 physical health summary subscale, and Patient Reported Outcomes Measurement Information Service (PROMIS) short form subscales for depression and anxiety.

**Results:**

The relationship between health literacy and patient activation was weak, but significant (r = 0.11, p<0.01). In models adjusted for participant characteristics, lower health literacy was associated with worse physical health (β = 0.13, p<0.001) and depression (β = −0.16, p<0.001). Lower patient activation was associated with worse physical health (β = 0.19, p<0.001), depression (β = −0.27, p<0.001) and anxiety (β-0.24, p<0.001).

**Conclusions:**

The most common measures of health literacy and patient activation are weakly correlated with each other, but also independently correlated with health outcomes. This suggests health literacy represents a distinct skill-based construct, supporting the Institute of Medicine’s definition. Deficits in either construct could be useful targets for behavioral intervention.

## Introduction

The field of health literacy has expanded over the last two decades [1], [2]. In a recent search of medical and public health literature, there were nearly 500 articles linking crude measures of literacy and numeracy skills to a range of health outcomes including physical and mental health and mortality [3]–[6].

This rapid growth has led to new definitions and interpretations of the term itself [2], [7]). In 2004, the Institute of Medicine accepted an earlier definition from Ratzan and Parker, clarifying health literacy to be *‘the degree to which individuals have the capacity to obtain, process, and understand basic health information and services needed to make appropriate health decisions’* [8]. The World Health Organization (WHO) expanded on this perspective, defining it as: *‘the cognitive and social skills which determine the motivation and ability of individuals to gain access to, understand and use information in ways which promote and maintain good health’* [9]. Perhaps most notable is that the WHO definition broadens the concept by including not only an individual’s health and healthcare ‘skill set’, but also their motivation or ‘mind set’ to engage in health promoting behaviors [9].

People who are motivated and confident in their ability to use their knowledge and skills are more likely to be active participants in maintaining and improving health. The term ‘patient and consumer activation’ has come to represent this, and is specifically defined as those who ‘…*have the motivation, knowledge, skills and confidence to make effective decisions to manage their health’* [10]. While measures such as locus of control and self-efficacy have been developed to measure aspects of activation, they tend to focus on one particular behavior. This led Hibbard and colleagues to develop a comprehensive measure of patient activation known as the Patient Activation Measure (PAM) [11]. This is considered to be a broader measure of activation that assesses general levels of activation for health self-management that is relevant across a wide range of health contexts. The PAM has been linked to several health processes and outcomes [12]–[15]. For example, in a sample of over 25,000 adult patients, Greene and Hibbard (2012) demonstrated associations with health limiting and health promoting behaviors, clinical indicators such as systolic blood pressure and costly healthcare utilization [16].

In a non-clinical national sample, the impact that patient activation could have on population health was demonstrated, with fewer than half (41%) of the population reaching the highest level of patient activation [12]. Importantly, patient activation varies by socio-economic status (SES) with individuals from lower SES backgrounds being less activated than higher SES groups [12], [16]. Interventions have been developed to improve patient activation, demonstrating that it is a manipulable construct that may be a route through which socioeconomic inequalities in health and healthcare could be reduced [17]–[19].

Despite interest in expanding the meaning of health literacy to include factors such as patient activation [9], [20], [21], existing measures of health literacy that have served as the foundation for the field for the past two decades do not directly assess these constructs; instead they involve reading and math tasks linked strongly to cognitive ability [22]–[25]. This is very different from measures such as the PAM, which assess an individual’s self-reported confidence in their ability to engage in self-management and health improvement. Definitions and conceptual models that combine the two under one umbrella term could be in danger of neglecting the unique contributions that health literacy and patient activation have to improving health outcomes.

To date, few studies have investigated the relationship of both patient activation and health literacy with health outcomes [26], [27]. For example, Greene and colleagues showed that patient activation was more closely aligned with health outcomes such as chronic disease self-management, while health literacy was more closely related to the ability to use information in health-relevant decisions [26]. These findings are however based on relatively small samples and have not examined physical or mental health as outcomes. The present study examined associations between health literacy, patient activation and physical and mental health. We hypothesized that in line with the IOM definition, health literacy is unique from patient activation and therefore an independent predictor of physical and mental health.

## Materials and Methods

### Study Design

The present study reports a secondary analysis of baseline cross-sectional data from the LitCog cohort. Details of the main outcomes from this data and detailed procedures are available elsewhere [25]. Briefly, this is a cohort of older American adults set up to observe the relationship between health literacy, cognitive ability and health outcomes.

### Participants

The baseline phase of LitCog recruited participants aged 55–74 from one primary care clinic and three federally qualified health centers in Chicago, Illinois. Recruitment ran from August 2008 through October 2010. A sample of 1768 eligible patients were reached by research staff and invited to participate in the study. Initial screening deemed 192 subjects as ineligible due to severe cognitive or hearing impairment, limited English proficiency, or not being connected to a clinic physician (defined as <2 visits in two years). In addition, 738 refused, 14 were deceased, and 20 were eligible but had scheduling conflicts. The final sample included 804 participants, giving a cooperation rate of 56% based on American Association for Public Opinion Research guidelines. A sub-sample of participants (n = 719) were also asked to complete a measure of patient activation. Data from these participants will be reported here. There were no missing data for gender or age. Participants were excluded from analyses if they had missing data for race, comorbidities, health literacy or patient activation (n = 22); giving a final sample for analyses of 697 patients.

### Procedure

Participation took place across two days, however all measures reported here were ascertained on the first day. Participants completed socio-demographic items, a health literacy measure, a measure of patient activation, and a series of health status measures. Participants were compensated $100 for their time. The Northwestern University Institutional Review Board approved the study procedures and all participants gave informed consent.

### Ethics

The study was approved by the Northwestern University’s Institutional Review Board. Participants provided written informed consent to participate in the study, which included permission to use the data for research.

### Measures

#### Health literacy

Health literacy was assessed using the Test of Functional Health Literacy in Adults (TOFHLA). The TOFHLA is an objective measure of health literacy which uses materials similar to those that patients encounter in healthcare situations [28]. The reading comprehension section includes 50 items that use the Cloze procedure; every fifth to seventh word in a passage is omitted and four multiple choice options are provided. The numeracy section includes 17 items to assess comprehension of labeled prescription vials, an appointment slip, a chart describing eligibility for financial aid, and an example of results from a medical test. During the development of the measure, validity was assessed by comparing associations with existing scales and a standard scoring system was formulated [28]. Participants are classified as having inadequate (0–59), marginal (60–74), or adequate (75–100) health literacy.

#### Patient activation

To assess activation, the shortened version of the PAM was used [29]. The PAM includes 13 items that assess self-reported knowledge, skill and confidence for health self-management and scores can range from 0–100. Example items include: *‘Taking an active role in my own health care is the most important factor in determining my health and ability to function’* and *‘I am confident that I can maintain lifestyle changes, like diet and exercise, even during times of stress’*. It is considered a non-illness-specific measure which captures aspects of motivation and engagement with health and self-management behaviors. The scale categorizes individuals as being in one of four stages of patient activation: i) believing that an active role is important in maintain and improving health ii) having confidence and knowledge to take action iii) taking action to maintain and improve one’s health iv) staying the course even under stress. The PAM has previously been validated against similar existing measures [29] and was found to be reliable in this study (α = .81).

#### Physical health

We assessed physical health using the SF-36 physical health summary subscale [30]. Scores are transformed into a 0–100 scale, with high scores indicating higher physical functioning. The SF-36 was found to be reliable (α = 0.90).

#### Mental health

Anxiety and depression were measured using the Patient Reported Outcomes Measurement Information Service (PROMIS) short form subscales [31]. Participants are given a score of 7–35 on the PROMIS-Anxiety scale and 8–40 on the PROMIS-Depression scale. High scores indicate greater anxiety and depression respectively. PROMIS-Anxiety (α = 0.91) and PROMIS-Depression (α = 0.91) were found to be reliable.

#### Participant characteristics

Participant characteristics were recorded. These included age, gender, marital status (married, unmarried) income (<$10,000, $10–24,999, $25–49,999, >$50,000) ethnicity (black, white, other) and comorbidities (0, 1–2, 3+).

### Statistical Analyses

One-way Analysis of Variance (ANOVA) was used to compare mean performance on the TOFHLA and PAM by participant characteristics. Associations between patient activation, health literacy, anxiety, depression and physical health were assessed using Pearson correlations. A series of multivariable linear regression models were conducted to examine the independent associations between health literacy, patient activation, and each of the physical and mental health status measures controlling for age, gender, race, and comorbidity. Standardized regression coefficients are reported throughout. Models were run first adding health literacy (Model 1) or patient activation (Model 2) alone in order to isolate the contributions of each, and then together (Model 3) to examine their combined effects. Outcomes are reported in the order of: (A) physical health, (B) anxiety and (C) depression. For example model A1 reports the association between health literacy and physical health, controlling for participant characteristics. F-tests were used to determine whether the variance explained by each of the models (R^2^) significantly changed with the addition of the other variable (i.e. Model 1 vs. Model 3 and Model 2 vs. Model 3). The Durbin-Watson statistic was used to investigate the assumption of independence. Normal probability (P-P) plots were used to investigate the normality of error terms and homoscedasticity was tested by observing the scatter plot of the residuals and the predicted value. These checks identified no violations of multiple regression assumptions. All statistical tests were one-tailed and a significance level of p<0.05 was set for all analyses. SPSS version 18.0 was used throughout.

## Results

The sample is described in Table 1. Participants were socially and economically diverse by years of schooling, household income, employment, marital status, and living situation. A third of the individuals had limited literacy skills according to the TOFHLA (inadequate 13.5%, 17.8% marginal), while the majority fell into the highest category of patient activation (level IV: 83.5%). The mean health literacy score was 76.45 (SD = 16.35) and the mean activation score was 78.95 (SD = 14.18). Participants average scores on the SF-36 (mean = 81.63, SD = 18.06), anxiety (mean = 15.38, SD = 5.87) and depression (mean = 13.16, SD = 6.23) were comparable to normative estimates.

**Table 1:** Sample Characteristics.

Characteristic	N	%
**Gender**		
Male	226	32.4
Female	471	67.6
**Age**		
55–59	221	31.7
60–64	213	30.6
65+	263	37.7
Marital status*		
Married	308	44.4
Unmarried	386	55.6
**Income**		
Missing	40	5.7
<$10,000	85	12.2
$10–24,999	132	18.9
$25–49,999	98	14.1
>$50,000	342	49.1
**Race**		
Black	309	44.3
Other	52	7.5
White	336	48.2
**Comorbidities**		
0	94	13.5
1–2	390	56
3+	213	30.6
**Health Literacy**		
Inadequate	94	13.5
Marginal	124	17.8
Adequate	479	68.7
**Patient Activation**		
Level 1	20	2.9
Level 2	27	3.9
Level 3	68	9.8
Level 4	582	83.5

(* = missing data).

### Health Literacy and Patient Activation

Higher levels of health literacy were found among females (p = 0.01, Table 2), white patients (p<0.001), and those with fewer comorbid conditions (p<0.001). Individuals that were older (p = 0.02), white (p = 0.02), and had fewer comorbidities (p<0.001) had higher levels of activation. There was a weak, positive correlation between health literacy and patient activation (r = 0.11, p = 0.005), indicating that individuals with higher levels of health literacy are more activated.

**Table 2:** Participant scores on the TOFHLA and PAM for socio-demographic subgroups.

	TOFHLA		Patient Activation Measure	
	Mean (SD)	p-value	Mean (SD)	p-value
**Gender**		.011		.828
Male	74.18 (19.59)		78.78 (13.25)	
Female	77.54 (14.43)		79.03 (14.61)	
**Age**		.054		.019
55–59	77.62 (16.15)		76.97 (15.16)	
60–64	77.61 (15.63)		78.92 (13.99)	
65+	74.53 (16.94)		80.62 (13.28)	
**Race**		.000		.024
Black	67.86 (18.01)		77.46 (15.38)	
Other	73.19 (9.29)		78.05 (13.84)	
White	84.85 (14.10)		80.45 (12.90)	
**Comorbidities**		.000		.000
0	82.19 (13.96)		81.44 (14.36)	
1–2	78.01 (14.57)		80.23 (13.63)	
3+	71.06 (18.81)		75.49 (14.51)	

### Physical Health

Both lower health literacy and patient activation were associated with worse physical health in univariate analyses (health literacy: r = 0.30, p<0.001; patient activation: r = 0.29, p<0.001). In multivariable models controlling for age, race and comorbidities, lower health literacy was related to worse physical health (Model A1: β = 0.15, p<0.001, Table 3). Lower patient activation was also significantly associated with worse physical health (Model A2: β = 0.20, p<0.001). When both were included in model A3, lower health literacy (β = 0.13, p<.001) and lower patient activation (β = 0.19, p<0.001) were independently associated with worse physical health. Female gender (β = −0.07, p<0.05), Black race (β = −0.12, p<0.01), and greater comorbidity (1–2: β = −0.16, p<0.001; 3+: β = −0.56, p<0.001) were also linked to worse physical health in model A3.

**Table 3:** Predicting Physical Health with Health Literacy and Patient Activation.

	Model A1	Model A2	Model A3
	β	β	β
**Female**	−.07*	−.06	−.07*
**Age**			
55–59	–	–	–
60–64	−.04	−.05	−.05
65+	.07	.02	.04
**Race**			
Black	−.12**	−.19***	−.12**
Other	−.01	−.03	−.01
White	–	–	
**Comorbidities**			
0	–	–	–
1–2	−.17***	−.16***	−.16***
3+	−.59***	−.57***	−.56***
**Health Literacy**	.15***	–	.13***
**PAM**	–	.20***	.19***

Note: * = p<.05; ** = p<.01; *** = p<.001.

Model A1 – Gender, age, race, comorbidities, health literacy (F_(8, 688)_ = 47.29***, R^2^_adj_ = .347).

Model A2 – Gender, age, race, comorbidities, PAM (F_(8, 688)_ = 52.04***, R^2^_adj_ = .370).

Model A3 – Gender, age, race, comorbidities, health literacy and PAM (F_(9, 687)_ = 48.74***, R^2^_adj_ = .382).

For each outcome, in order to test whether including both health literacy and patient activation significantly improved the explanatory power of Models A1 and A2, the R^2^ change statistic was observed. There were significant differences between models A1 and A3 (R^2^ change = 0.04; F(1,687) change = 39.28, p<0.001) and between models A2 and A3 (R^2^ change = 0.01; F(1, 687) change = 14.25, p<0.001), indicating that including both health literacy and patient activation significantly improved explanatory power in the physical health outcome compared to either one alone. Interactions were tested but found to be non-significant.

### Mental Health

#### Anxiety

Lower health literacy and lower patient activation were both significantly associated with greater anxiety in univariate analyses (health literacy: r = −0.11, p = 0.005; patient activation: r = −0.29, p<0.001). In multivariable analyses controlling for age, race and comorbidities, lower health literacy was independently associated with greater anxiety (Model B1: β = −0.09, p = 0.035; Table 4). Lower patient activation was also significantly associated with higher levels of anxiety (Model B2: β = −0.24, p<0.001). When both were entered in the same model (model B3), patient activation maintained its level of significance (β = −0.24, p<0.001), while health literacy did not (β = −0.07, p = 0.077), but there was very little attenuation of the effect size from Model B2 to Model B3. Other significant predictors of anxiety symptoms in model B3 included age (65+: β = −0.16, p<0.001) and comorbidity (1–2: β = 0.12, p<0.05; 3+: β = 0.32, p<0.001).

**Table 4:** Predicting Mental Health Outcomes with Health with Health Literacy and Patient Activation.

	PROMIS Anxiety			PROMIS Depression		
	Model B1	Model B2	Model B3	Model C1	Model C2	Model C3
	β	β	β	β	β	β
**Female**	.06	.06	.07	.01	−.01	.01
**Age**						
55–59	–	–	–	–	–	–
60–64	−.07	−.05	−.05	−.04	−.02	−.02
65+	−.19***	−.15***	−.16***	−.20***	−.14***	−.16***
**Race**						
Black	−.07	−.04	−.07	−.02	.05	−.03
Other	.07	.07*	.06	.08*	.10**	.08*
White	–	–	–	–	–	–
**Comorbidities**						
0	–	–	–	–	–	–
1–2	.13*	.12*	.12*	.11*	.11*	.10*
3+	.36***	.33***	.32***	.39***	.36***	.34***
**Health Literacy**	−.09*	–	−.07	−.17***	–	−.16***
**PAM**	–	−.24***	−.24***	–	−.27***	−.27***

Note: * = p<.05; ** = p<.01; *** = p<.001.

PROMIS Anxiety:

Model B1 – Gender, age, race, comorbidities, health literacy (F_(8, 688) = _11.59***, R^2^_adj_ = .109).

Model B2 – Gender, age, race, comorbidities, PAM (F_(8, 688) = _17.33***, R^2^_adj_ = .158).

Model B3 – Gender, age, race, comorbidities, health literacy and PAM (F_(9, 687) = _15.80***, R^2^_adj_ = .161).

PROMIS Depression:

Model C1 – Gender, age, race, comorbidities, health literacy (F_(8, 688) = _19.05***, R^2^_adj_ = .172).

Model C2 – Gender, age, race, comorbidities, PAM (F_(8, 688) = _25.92***, R^2^_adj_ = .223).

Model C3 – Gender, age, race, comorbidities, health literacy and PAM (F_(9, 687) = _25.23***, R^2^_adj_ = .239).

Adding patient activation to anxiety Model B1, which included health literacy alone, significantly improved its explanatory power (R^2^ change = 0.05; F(1, 687) change = 43.73, p<0.001). However, there was no significant difference between Models B2 and B3 (R^2^ change = 0.004; F(1, 687) change = 3.15, p = 0.077), indicating that health literacy did not explain a significant amount of additional variance in anxiety after patient activation had been entered in the model. We also tested for an interaction between health literacy and patient activation, and this was not significant.

#### Depression

Similar to anxiety, lower health literacy and patient activation were both significantly related to more depressive symptoms in univariate analyses (health literacy: r = −0.22, p<0.001; patient activation: r = −0.34, p<0.001). In multivariable analyses controlling for age, race and comorbidities, lower health literacy was independently associated with worse depression (Model C1: β = −0.17, p<0.001, Table 4). Lower levels of patient activation were also associated with worse depression (Model C2: β = −0.27, p<0.001). When both were included in model C3, lower health literacy remained a predictor of depression (β = −0.16, p<0.001), as did lower patient activation (β = −0.27, p<0.001). In model C3, age (65: β = −0.16, p<0.001) and comorbidity (1–2: β = 0.10, p<0.05; 3+: β = 0.34, p<0.001) were associated with depression.

There was a significant difference between models C1 and C3 (R^2^ change = 0.07; F(1, 687) change = 61.28, p<0.001), and also between models C2 and C3 (R^2^ change = 0.02; F(1, 687) change = 15.35, p<0.001), indicating that the explanatory power of either health literacy or patient activation was significantly improved when the other was included. No interactions were found in the depression models.

## Discussion

In this sample of older American adults, health literacy and patient activation were independently associated with depression and physical health when included in the same statistical model. Health literacy was not significantly associated with anxiety, and patient activation was the stronger predictor of the two measures for all health outcomes. These findings are in line with previous general population studies [5], [6], [13] and studies in condition-specific groups [32]. Importantly, health literacy and patient activation were often more strongly associated with health outcomes than known correlates such as ethnicity and age. There was a weak association between health literacy and patient activation and very little attenuation occurred across models when entering patient activation and health literacy in tandem. In support of the IOM definition, health literacy and patient activation appear to be two independent constructs, influencing health via different pathways.

Collectively, these findings suggest health literacy, as it is currently measured by the most widely used assessment tool [4], is predominantly a skills-based construct that has not included motivational elements. It could be argued that this definition is taking a broader approach to conceptualizing health literacy [2], [20] that is not bound to current and often criticized measures. However, the widening gap between how the construct is currently defined and assessed for research purposes should be recognized. The continued use of a broader health literacy definition challenges behavioral science researchers to develop new methods of assessment. One possibility would be to develop a brief psychometric measure that includes elements of both cognitive and motivational constructs. Although routine collection of health literacy data, especially for clinical purposes, has been questioned [33], [34], a measure combining basic health literacy skills and patient activation could be attractive to clinicians attempting to identify the specific needs of their patients.

Our findings have implications both for the individual treatment of patients, and for large-scale health interventions that affect the public more widely. For example clinicians attending to the health literacy needs of their patients by simplifying treatment regimens and clarifying instructions may be inadvertently missing opportunities to activate their patients. The assumption that an individual with the ‘skill set’ for how to act will automatically adhere to instructions, ignores the ‘mind set’ factors that underpin behavior change. Similarly, a focus on patient activation may fail to acknowledge the difficulties faced by those lacking the adequate ‘skill set’, despite being activated to self-manage their health. From a public health perspective, patient-centered interventions to improve health outcomes may be best served by incorporating elements of both health literacy and activation into their design and evaluation. The strong associations of health literacy [35] and patient activation [12], [16] with socioeconomic status suggest individual and public health approaches that address these issues may concomitantly reduce health disparities in health and healthcare. However prospective studies that provide a firmer basis to assume a causal relationship are needed to make this step.

Mechanisms have been suggested through which health literacy and patient activation could be associated with mental and physical health. The strongest evidence suggests individuals with low health literacy find accessing and understanding health information more difficult [4], [36], which can result in disparities in health knowledge [37]–[40], fewer disease prevention behaviors [41]–[44] and inconsistent medication adherence [45]. In contrast, even if individuals have the skills to access health information easily, those with low levels of patient activation may still feel they are less able to self-manage their health, with evidence suggesting they have lower confidence in help-seeking, are more passive in communicative situations, less proactive in changing current health behaviors such as diet and exercise and less likely to be open to new ways of solving health problems [12], [13]. Ultimately, individuals that are deficient in either or both constructs are at greater risk of experiencing poorer health, but there may be different ways of intervening depending on the specific needs of the individual.

A strength of this study was the use of a large, socioeconomically diverse general population sample recruited from multiple sites, including academic and community-based services. Gold standard versions of the most commonly used measures of each of the dependent and independent variables were also used. Furthermore, previous models have typically only included either health literacy or patient activation. This study is also among the first to demonstrate the individual and combined effects of these constructs on important health outcomes, testing a hypothesis that was generated by the discrepancy between key health literacy definitions.

This study had limitations. The cross-sectional nature of the study prohibits causal inferences. Furthermore, estimates of associations were drawn from a sample that had higher levels of both activation and health literacy than normative estimates [12], [46], which could lead to underestimating the true strength of the relationships. It is possible that the relationship between patient activation and health literacy would be stronger among lower literacy groups, however further research is required in order to investigate this hypothesis. The models tested did not attempt to control for the wide range of additional factors that can contribute to health. However the primary aim of this paper was not to explain variance over and above known risk factors, but rather to use available data to test the definitions of health literacy put forth by two major health organizations. Future research may wish to observe these relationships while controlling more stringently for known covariates of physical and mental health. Finally, the exclusion of patients that had not seen a regular physician for two years may make the sample less generalizable to the wider population but this was necessary in order to retain and track individuals for future follow up.

The next step for research in this field would be to investigate whether similar effects are apparent in different health domains, such as complex health tasks, self-management and healthcare utilization. This will permit researchers to determine whether the relative importance of each construct varies in different circumstances, allowing specific policy recommendations to be made for each situation. As discussed previously, these findings strongly suggest there may be scope for behavioral scientists to develop a comprehensive measure that assesses both basic skills and activation within a single brief tool.

In conclusion, health literacy and patient activation are weakly correlated with each other, and also make independent contributions to health. Deficits in either domain could be useful targets for behavioral intervention. New measurement strategies are needed to evaluate both constructs and a combined approach may be attractive not only to researchers but also to clinicians who wish to identify patients who need further support. In the meantime, we recommend that health literacy and patient activation be treated as distinct and important constructs warranting assessment in public health and behavioral science research.
